# Comparison of clinical features between *Chlamydia psittaci* and *Legionella*

**DOI:** 10.3389/fmed.2025.1653394

**Published:** 2025-11-12

**Authors:** Jiamei Chen, Libing Yang, Yuni Liu, Jianliang Zhou, Yongzhong Li, Jin Wang, Yixiang Zheng

**Affiliations:** 1 Department of Infectious Diseases, Hunan University of Medicine General Hospital, Huaihua, Hunan, China; 2 Department of Gastroenterology, Yiyang Third People’s Hospital, Yiyang, Hunan, China; 3 Department of Intensive Care Medicine, Hunan University of Medicine General Hospital, Huaihua, Hunan, China; 4 Department of Infectious Diseases, The First Affiliated Hospital of Xiangya Hospital, Changsha, Hunan, China

**Keywords:** *Chlamydia psittaci* pneumonia, *Legionella* pneumonia, clinical features, next-generation sequencing, diagnosis

## Abstract

**Objectives:**

Traditional diagnostic methods have difficulty distinguishing between *Chlamydia psittaci* (*C. psittaci*) pneumonia and *Legionella* pneumonia (*L*. pneumonia). This study aims to delineate the differences between *C. psittaci* pneumonia and *L. pneumonia*.

**Methods:**

This retrospective analysis included 71 cases of *C. psittaci* pneumonia and 21 cases of *L*. pneumonia, all confirmed via next-generation sequencing (NGS). We systematically collected and compared data on clinical characteristics, laboratory findings, chest CT imaging, bronchoscopic observations, and prognostic outcomes between the two groups.

**Results:**

In the *C. psittaci* pneumonia cohort, 64 patients (91.4%) had an opportunity to contact with poultry, with a maximum temperature of mean 39.6 °C. Additionally, 23 patients (32.4%) experienced dyspnea, and 57 patients (80.3%) exhibited relative bradycardia. Compared to patients with *L*. pneumonia, those with *C. psittaci* pneumonia had lower leukocyte counts, neutrophil counts, mononuclear cell counts, systemic inflammatory response index (SIRI), and urea levels, while lymphocyte-to-monocyte ratio (LMR), glutamic-pyruvic transaminase (ALT), glutamic oxaloacetic transaminase (AST), and creatine kinase (CK) levels were elevated. Next-generation sequencing (NGS) plays a crucial role in the early diagnosis of infectious pathogens. Multivariate analysis revealed differences in underlying diseases, residing in countryside, relative bradycardia, and LMR between the two groups.

**Conclusion:**

Several characteristics aid in differentiating *C. psittaci* pneumonia from *L*. pneumonia, including exposure to poultry, relative bradycardia, some infection indicators, ALT, AST, and CK. NGS addresses the limitations of traditional diagnostic methods. The early application of NGS facilitates the diagnosis of atypical pneumonia. Multivariate regression analysis suggested that underlying diseases, residing in countryside, relative bradycardia, and LMR is significant in differentiating *C. psittaci* pneumonia and *L*. pneumonia.

## 1 Introduction

Atypical bacterial pneumonia arises from infection with atypical pathogens that cannot be detected using Gram staining and are challenging to culture using standard methods. Common pathogens include *Mycoplasma pneumoniae*, *Chlamydia pneumoniae*, *Legionella pneumophila*, and *Coxiella burnetii* (Q fever) (1, 2). The predominant clinical manifestations encompass systemic and respiratory symptoms. Systemic symptoms, such as headache, low-grade fever, and general malaise, were more pronounced than respiratory symptoms, with the primary respiratory manifestation being a persistent dry cough. *Chlamydia psittaci(C. psittaci) Chlamydia psittaci* causes a rare form of pneumonia, comprising approximately 1% of community-acquired pneumonia cases, and is difficult to diagnose due to its nonspecific presentation (3, 4). The nonspecific nature of *C. psittaci* pneumonia’s clinical presentation, along with the limited accuracy of conventional diagnostic methods, makes diagnosis particularly challenging (5). Research indicates that *Legionella* spp. is among the four most common microbial causes of CAP-related hospitalizations (6). In patients with severe CAP requiring hospitalization, 2 to 15% are infected with *Legionella* (7, 8). The rarity and slow growth of *Legionella* in non-selective culture-based assays further complicate the diagnosis of such infections (9). The clinical manifestations and examination results of these two pathogens share many similarities. Common symptoms, which range from mild to severe and lack specificity, include fever, chills, cough, expectoration, dyspnea, fatigue, and some extrapulmonary manifestations (10). Research has demonstrated that infections caused by atypical pathogens, such as *Legionella* and *Chlamydia psittaci*, can affect multiple organ systems, complicating the differential diagnosis between *L*. pneumonia and *C. psittaci* pneumonia.

Next,-generation sequencing (NGS) has been extensively utilized due to its rapid and precise capabilities (11). Although various studies have individually reported on the clinical characteristics of *C. psittaci* pneumonia and *Legionella* pneumonia, there is a dearth of literature that directly compares them. This study aims to elucidate the clinical features, laboratory findings, and imaging results associated with *C. psittaci* pneumonia and *L*. pneumonia, offering clinicians essential insights for differentiating these conditions.

## 2 Patients and methods

### 2.1 Patient

A retrospective, single-center study was conducted at the Hunan University of Medicine General Hospital from March 2019 to January 2025. All included patients met the following criteria: (1) Diagnosis of atypical community-acquired pneumonia (CAP) in accordance with current clinical guidelines (12). Next-generation sequencing (NGS) diagnosis was performed using bronchoalveolar lavage fluid, lung tissue samples, blood specimens, or sputum samples. The exclusion criteria included patients with AIDS, tuberculosis, silicosis, active malignant tumors, and other severe underlying lung diseases.

### 2.2 Study design

The clinical characteristics, laboratory examination results, and findings from chest computed tomography (CT) scans laboratory test results, bronchoscopic observations, and prognosis of each patient at the time of admission were systematically extracted from the hospital’s electronic medical record system. Specifically: (1) clinical characteristics included demographic information such as age and gender, residential area (urban vs. rural), underlying conditions (hypertension, coronary artery disease, diabetes mellitus, viral hepatitis, and immunosuppressive therapy), as well as clinical symptoms and signs. (2) laboratory tests comprised a comprehensive panel of blood tests, assessments of liver and kidney function, electrolyte levels, traditional inflammatory markers include C-reactive protein (CRP), procalcitonin (PCT), erythrocyte sedimentation rate (ESR), and interleukin-6 (IL-6), while novel markers include the neutrophil-to-lymphocyte ratio (NLR), monocyte-to-lymphocyte ratio (MLR), and lymphocyte-to-white blood cell ratio (LWR), platelet-to-lymphocyte ratio (PLR), systemic immune-inflammation index (SII), plasma D-dimer concentrations, and myocardial enzyme levels. (3) the chest CT findings provided a detailed assessment of the extent of pulmonary involvement, including the presence of pleural or pericardial effusions, pleural thickening, and mediastinal lymphadenopathy. (4) Bronchoscopic observations revealed mucosal erythema, edema, and secretions.

### 2.3 Next-generation sequencing detection method

Gene sequencing enables the direct acquisition of DNA from all microorganisms present in clinical samples, facilitating the study of microbial DNA composition and community function through genomics (13, 14). This technique is characterized by its rapidity, accuracy, and objectivity (11). It has been extensively applied in the diagnosis and treatment of infectious diseases, demonstrating significant advantages in identifying rare pathogens (15).

### 2.4 Data analysis

Data analysis was conducted using SPSS software (version 26.0). Continuous variables with normal distribution are reported as means ± standard deviations, whereas those with non-normal distribution are presented as medians with interquartile ranges (IQR). Categorical variables are summarized using frequencies and percentages. For the purpose of statistical analysis, continuous data exhibiting a normal distribution were evaluated using the independent samples *t*-test, while non-normally distributed continuous data were analyzed via the Mann–Whitney U test. Categorical data were assessed using either the *χ*^2^ test or Fisher’s exact test, as deemed appropriate. All *p*-values reported are two-sided, with statistical significance defined at *p* < 0.05.

## 3 Results

### 3.1 Clinical characteristics

In this retrospective analysis, clinical data were systematically gathered from 92 patients diagnosed using Next-Generation Sequencing (NGS), which included 71 cases of *C. psittaci* pneumonia and 21 cases of *L*. pneumonia.

Within the *C. psittaci* pneumonia cohort, a majority of patients (91.4%) resided in rural areas, with many having neighbors who kept poultry. This proportion was significantly higher compared to the *L*. pneumonia group (47.6%). All patients with psittaci pneumonia presented with fever, and their maximum recorded temperatures were higher than those observed in patients with *Legionella* pneumonia. Furthermore, patients infected with *C. psittaci* demonstrated a significantly greater incidence of relative bradycardia compared to those with *L. pneumophila* (80.3% versus 23.8%, *p* = 0.000). furthermore, multivariate analysis revealed differences in Underlying diseases, Residing in countryside and Relative bradycardia between the *Legionella* pneumonia and *Chlamydia psittaci*. The General clinical data, clinical manifestations and Clinical signs are shown in Table 1.

**Table 1:** General clinical data, clinical manifestations and clinical signs of patients with *C. psittaci* pneumonia and *L*. pneumonia.

Univariate analysis				Multivariate analysis		
Clinical features	*Chlamydia psittaci* pneumonia (*n* = 71)	*Legionella* pneumonia (*n* = 21)	*p* value	OR	95% CI	*p* value
General clinical data						
Gender (male/female)	46/25	16/5	0.328			
Age (years)	62.410 ± 10.505	62.620 ± 13.673	0.94	1.011	0.965–1.059	0.650
Underlying diseases	35 (49.300%)	15 (71.400%)	0.074	0.286	0.092–0.896	0.032
Residing in countryside	64 (91.400%)	10 (47.600%)	0	0.028	0.002–0.368	0.007
Clinical manifestations						
Maximum temperature	39.600 (39.000, 40.000)	39.000 (38.750, 39.600)	0.003	0.888	0.348–2.264	0.803
Cough	54 (76.100%)	18 (85.700%)	0.548			
Coughing up phlegm	40 (56.300%)	16 (76.200%)	0.102			
Chills	33 (46.500%)	6 (28.600%)	0.145			
Anhelation	23 (32.400%)	14 (66.700%)	0.005	4.041	0.562–29.077	0.165
Clinical signs						
Rough breathing sound	41 (57.700%)	12 (57.100%)	0.961			
Low breathing sound	16 (22.500%)	4 (19.000%)	1			
Wet rales	44 (62.000%)	12 (57.100%)	0.69			
Relative bradycardia	57 (80.300%)	5 (23.800%)	0	0.053	0.005–0.579	0.016
Severe pneumonia	25 (35.200%)	7 (33.300%)	0.874			

### 3.2 Laboratory parameters

Among patients with *C. psittaci* pneumonia, there were increased levels of the lymphocyte/monocyte ratio (LMR), alanine aminotransferase (ALT), aspartate aminotransferase (AST), and creatine kinase (CK). Conversely, in the group with *L. pneumophila* pneumonia, patients exhibited higher levels of white blood cells (WBC), neutrophil count, monocyte count, and systemic inflammatory response index (SIRI), and. Furthermore, multivariate analysis revealed differences in LMR between the *Legionella* pneumonia and *Chlamydia psittaci*. The laboratory parameters are detailed in Table 2.

**Table 2:** Laboratory parameters of patients with *C. psittaci* pneumonia and *L*. pneumonia.

Univariate analysis				Multivariate analysis		
Aboratory parameters (Unit)	*Chlamydia psittaci* pneumonia (*n* = 71)	*Legionella* pneumonia (*n* = 21)	*p* value	OR	95%CI	*p* value
WBC (*109 /L)	8.170 ± 2.651	11.771 ± 6.234	0.017	2.268	0.334–15.383	0.402
Hb(g/l)	113.040 ± 21.080	110.760 ± 17.810	0.654			
PLT (*109 /L)	145.000 (117.000,219.000)	131.000(92.500, 187.000)	0.418			
N(%)	88.500 (83.400, 93.400)	88.400 (71.800, 94.450)	0.11			
N (*109 /L)	7.312 ± 2.851	10.511 ± 6.144	0.047	0.420	0.062–2.865	0.376
M (*109 /L)	0.270 (0.160, 0.465)	0.496 ± 0.344	0.04	0.001	0.000–2.502	0.086
L (*109 /L)	0.485 (0.300, 0.738)	0.713 ± 0.515	0.353			
NLR	13.330 (7.423, 26.115)	18.694 (7.978, 35.695)	0.21			
LMR	1.852 (1.305,2.595)	1.187 (0.881,2.146)	0.035	0.459	0.233–0.903	0.024
LWR	0.067 (0.036, 0.109)	0.067 ± 0.058	0.221			
PLR	343.791 (193.083, 467.659)	271.296 (167.040,418.867)	0.421			
SII	2008.460 (982.488, 3980.680)	2262.028 (1090.996,6968.196)	0.692			
SIRI	3.614 (2.170, 6.944)	9.473 ± 7.187	0.004	0.823	0.600–1.131	0.230
CRP(mg/l)	164.900 (121.520, 216.100)	162.112 ± 119.297	0.262			
PCT(ug/l)	0.940 (0.400, 3.260)	0.910(0.285, 2.830)	0.46			
ESR(mm/h)	74.740 ± 34.056	62.470 ± 30.847	0.19			
IL-6(pg/ml)	95.360 (62.790, 342.100)	214.600 (57.765, 933.925)	0.222			
D-dimer(μg/ml)	2.270 (1.230, 5.010)	1.810 (0.675, 4.880)	0.397			
ALT(IU/L)	47.000 (24.000, 94.000)	21.000 (15.000, 40.500)	0.004	1.018	0.985–1.052	0.302
AST(IU/L)	77.000 (38.000, 141.000)	25.000 (14.500, 46.500)	0	1.006	0.976–1.038	0.685
ALB(g/L)	31.727 ± 6.120	31.790 ± 5.163	0.966			
TBIL(umol/L)	12.600 (8.800, 20.300)	11.900 (6.850, 27.150)	0.612			
DBIL(umol/L)	6.000 (4.000, 9.800)	5.900 (2.300, 12.650)	0.561			
CREA-S(umol/L)	87.000 (72.000, 106.000)	94.000 (74.000, 174.000)	0.254			
UA(umol/L)	196.000 (155.000, 270.500)	237.000 (161.000, 371.500)	0.176			
LDH(U/L)	321.000 (255.000, 494.000)	310.480 ± 151.085	0.077			
CK(U/L)	154.000 (82.000, 340.000)	72.000 (32.000, 136.500)	0.008	1.000	0.998–1.002	0.873
CK-MB(IU/L)	13.000 (8.000, 20.000)	15.810 ± 9.108	0.716			
MYO(ug/L)	120.000 (69.000, 443.000)	109.000 (60.500, 336.000)	0.391			
K (mmol/L)	3.769 ± 0.447	4.139 ± 1.055	0.132			
Na (mmol/L)	136.326 ± 6.114	137.481 ± 4.931	0.431			
Cl(mmol/L)	103.450 (99.900, 107.600)	103.324 ± 5.376	0.631			
Ca (mmol/L)	1.970 (1.910, 2.060)	2.031 ± 0.169	0.07			
Corrective Ca (mmol/L)	2.026 ± 0.145	2.077 ± 0.185	0.188			

*WBC, white blood cell; PLT, platelets; N%, percentage of neutrophils; NLR: Neutrophil to lymphocyte ratio; MLR, monocyte/lymphocyte ratio; LMR, lymphocyte/monocyte ratio; LWR, lymphocyte/leukocyte ratio; PLR, platelet/ lymphocyte ratio; SII: platelet *Neutrophil/lymphocyte ratio; CRP, C-reactive protein; PCT, procalcitonin; ESR, erythrocyte sedimentation rate; IL-6, interleukin-6; ALT, alanine aminotransferase; AST, aspartate aminotransferase; TBIL, total bilirubin; DBIL, direct bilirubin; CREA-S, serum creatinine; UA, uric acid; LDH, lactate dehydrogenase; CK, creatine kinase; CK-MB, creatine kinase isoenzyme; MYO, myoglobin; ALB, albumin.

### 3.3 Chest computed tomography and bronchoscopic observations

All patients underwent chest computed tomography (CT) examinations. Among those with *C. psittaci* pneumonia, 31 patients (43.7%) displayed unilateral lung lesions, whereas in the *L. pneumophila* pneumonia group, only 3 patients (14.3%) showed unilateral lung lesions. The chest CT findings are presented in Table 3.

**Table 3:** Chest computed tomography and bronchoscopic observations of patients with *C. psittaci* pneumonia and *L*. pneumonia.

Inspection results	*Chlamydia psittaci* pneumonia (*n* = 71)	*Legionella* pneumonia (*n* = 21)	*p* value
Chest computed tomography			
Unilateral lesion	31 (43.700%)	3 (14.300%)	0.014
Irregular and inconsistent shadows	66 (94.300%)	19 (90.500%)	0.619
Pleural effusion	48 (67.600%)	15 (71.400%)	0.740
Pericardial effusion	20 (28.200%)	5 (23.800%)	0.693
Bronchial inflation sign	6 (8.500%)	1 (4.800%)	1.000
Pleural thickening	36 (50.700%)	9 (42.900%)	0.527
Mediastinal lymphadenopathy	31 (43.700%)	8 (38.100%)	0.650
Pulmonary nodular changes	8 (11.400%)	2 (9.500%)	1.000
Atelectasis	1 (2.900%)	1 (4.800%)	1.000
Bronchoscopic manifestations			
Bronchial mucosa was obviously congested and swollen	64 (95.500%)	20 (100.000%)	1.000
Less secretion	42 (62.700%)	13 (65.000%)	0.851

Regarding bronchoscopic observations, 20 patients in the *C. psittaci* pneumonia group and 64 patients in the *L. pneumophila* pneumonia group underwent this procedure. The bronchoscopic findings are also included in Table 3.

### 3.4 Treatment and recovery

Prior to diagnosis, the majority of patients received empirical treatment with a beta-lactam antibiotic, which was ultimately found to be ineffective. Subsequently, samples of bronchoalveolar lavage fluid (BALF), blood, lung tissue, or sputum were collected for next-generation sequencing (NGS) analysis to confirm the diagnosis and guide appropriate treatment changes. In the group with *C. psittaci* pneumonia, 51 patients (71.8%) received oxygen therapy; of these, 42 patients utilized a nasal cannula for oxygen inhalation, while 18 patients required ventilator-assisted breathing. Following treatment modifications, 65 patients (91.5%) exhibited gradual clinical and laboratory improvement. In the *L. pneumophila* pneumonia group, 20 patients (95.2%) received oxygen therapy; among them, 8 patients used a nasal cannula for oxygen inhalation, and 5 patients were treated with ventilator-assisted breathing. After treatment adjustments, 14 patients (66.7%) demonstrated gradual clinical and laboratory improvement.

## 4 Discussion

In atypical pneumonia, both *L. pneumophila* and *C. psittaci* can affect multiple organ systems, and their clinical characteristics are largely the same (16, 17). Research has previously indicated that *C. psittaci* pneumonia is chiefly transmitted through the inhalation of aerosols containing dried secretions or waste from the respiratory systems of infected birds, also through bird bites, contact through the mouth, touching feathers and tissues of infected birds (18, 19). *Legionella* species are commonly found in various natural environments, including water sources and soil, comprising 58 species and 3 subspecies (20). Patients with *L*. pneumonia often have non-specific epidemiological histories, such as recent bathing, travel, or cruise ship exposure (16, 21). In the present study, both groups predominantly consisted of elderly male patients with pre-existing conditions. *C. psittaci* pneumonia was more frequently observed in rural areas, where individuals have increased exposure to poultry.

Previous research has demonstrated that common symptoms of *C. psittaci* pneumonia and *L. pneumophila* pneumonia include fever, cough, expectoration, dyspnea, and chills (22). The pneumonia caused by *C. psittaci* is known for respiratory and systemic symptoms, including muscle pain, headaches, and central nervous system involvement. Fever, dyspnea, dry cough, and headache are the most prevalent symptoms, common physical indicators are dry and wet rales in the lungs, paired with a relatively bradycardic pulse (23, 24). *L. pneumophila* pneumonia typically presents with a subacute onset, with over half of the patients experiencing fatigue, weakness, myalgia, chills, and high fever, often accompanied by a dry cough and chest pain. Some patients may also exhibit hemoptysis, nausea, vomiting, or abdominal diarrhea, and as lung lesions progress, severe cases may develop dyspnea (25). In this study, the majority of patients exhibited fever, cough, expectoration, rough breathing sounds, and wet rales. Patients with *C. psittaci* pneumonia demonstrated higher body temperatures, a greater incidence of dyspnea, and relative bradycardia. Multivariate analysis revealed most patients with *Legionella* pneumonia have underlying diseases, while those with *Chlamydia psittaci* pneumonia mostly residing in countryside and have a relative bradycardia.

Previous research has demonstrated that *C. psittaci* pneumonia may lead to normal or slightly elevated white blood cell counts (26), as well as increased levels of CRP and ESR in patients (27, 28). In contrast, leukocyte count and procalcitonin (PCT) levels are significantly elevated in cases of *L. pneumophila* pneumonia (29). New research has validated the effectiveness of inflammatory markers such as LMR, NLR, LWR, PLR, SIRI, and SII in accurately and sensitively reflecting the body’s inflammation levels, facilitating the evaluation of various diseases and their prognoses (30, 31). In the present study, the percentage of neutrophils, CRP, ESR, interleukin-6 (IL-6), and other inflammatory markers were significantly elevated in the majority of patients. Importantly, patients with *C. psittaci* pneumonia had lower levels of white blood cells, neutrophils, monocytes, SIRI, and urea than those with *L. pneumophila* pneumonia. Conversely, LMR, ALT, AST, and CK levels were higher in patients with *C. psittaci* pneumonia. Pulmonary imaging findings for *C. psittaci* pneumonia and *L. pneumonia* are similar, exhibiting diverse yet comparable features, including perihilar ground-glass opacities and unilateral or multilobar consolidation. In this study, *C. psittaci* pneumonia and *L. pneumophila* pneumonia were examined. Multivariate analysis showed that the LMR value of *Chlamydia psittaci* pneumonia was relatively higher.

Pulmonary imaging characteristics of *C. psittaci* pneumonia and *L. pneumophila* pneumonia exhibit similarities, with both conditions presenting varied findings such as perihilar ground-glass opacities and unilateral or multilobar consolidation (32, 33). In the present study, *C. psittaci* pneumonia and *L. pneumophila* pneumonia predominantly manifested as irregular and inconsistent shadows accompanied by pleural thickening.

The therapeutic agents deemed effective for *C. psittaci* pneumonia include tetracyclines, quinolones, and macrolides (34, 35). while *L. pneumophila* pneumonia is effectively treated with macrolides, rifampicin, and third-generation quinolones. *C. psittaci* pneumonia generally has a favorable prognosis, with a mortality rate as low as 1% when timely and appropriate treatment is administered, but this rate can increase to 10–20% in the absence of such treatment (36). In contrast, studies indicate that *L. pneumophila* pneumonia has a mortality rate of approximately 7% (37, 38). In this study, 65 patients (91.5%) with *C. psittaci* pneumonia achieved cure following treatment with doxycycline, whereas 14 patients (66.7%) with *Legionella* pneumonia were cured after receiving macrolide therapy.

This study presents several limitations. Firstly, the primary limitation is the inclusion of only 21 patients diagnosed with *L. pneumophila* pneumonia; a larger sample size would enhance the robustness of multivariate analyses. Secondly, the study was conducted as a single-sample retrospective analysis, and alternative diagnostic methods were not employed to confirm the diagnosis. Thirdly, urinary antigen testing for *L. pneumophila* pneumonia was not conducted.

## 5 Conclusion

Although the clinical profile of patients suffering from *C. psittaci* pneumonia and *L. pneumophila* pneumonia are similar, inflammatory markers were elevated in patients with *L. pneumophila* pneumonia. Additionally, patients with *C. psittaci* pneumonia demonstrated better recovery outcomes compared to those with *L. pneumophila* pneumonia. The early application of next-generation sequencing (NGS) improved the detection rates of both *C. psittaci* pneumonia and *L. pneumophila* pneumonia, facilitated treatment guidance, and enhanced patient prognosis. Multivariate regression analysis suggested that underlying diseases, residing in countryside, relative bradycardia, and LMR is significant in differentiating *C. psittaci* pneumonia and *L*. pneumonia.

## Data Availability

The original data of this article provided by the authors without inappropriate reservations.
